# Personality Traits Modulate the Impact of Emotional Stimuli During a Working Memory Task: A Near-Infrared Spectroscopy Study

**DOI:** 10.3389/fnbeh.2020.514414

**Published:** 2020-09-17

**Authors:** Masaaki Sugi, Satoshi Sakuraba, Hirotada Saito, Mitsunori Miyazaki, Susumu Yoshida, Tatsuhiro Kamada, Shinya Sakai, Daisuke Sawamura

**Affiliations:** ^1^Department of Rehabilitation, Tokeidai Memorial Hospital, Hokkaido, Japan; ^2^Department of Rehabilitation Sciences, Health Sciences University of Hokkaido, Hokkaido, Japan; ^3^Department of Functioning and Disability, Faculty of Health Sciences, Hokkaido University, Hokkaido, Japan

**Keywords:** personality trait, emotional stimuli, dorsolateral prefrontal cortex, near-infrared spectroscopy, dual *n*-back task

## Abstract

The purpose of the present study was to examine the influence of personality traits on the impact of emotional stimuli focusing on *n*-back task performance and brain activity changes. Previous neuroimaging studies have reported that individual differences in emotional processing can be attributed to personality traits, which is linked to the hemisphere-specific activity of the dorsolateral prefrontal cortex (DLPFC) in response to emotional stimuli. Thirty right-handed healthy young male participants were recruited in this study and classified into two groups, the behavioral inhibition system (BIS) group and behavioral activation system (BAS) group, based on their scores on the BIS/BAS scale. Participants saw six emotional images (two each with negative, neutral, and positive valence), which were selected from the International Affective Picture System and validated in a preliminary experiment. Then, a dual 2-back task that simultaneously employed auditory-verbal and visuospatial stimuli was conducted. Additionally, the concentration of oxygenated hemoglobin (Oxy-Hb) changes in the DLPFC was measured during the image presentation and dual 2-back task by near-infrared spectroscopy (NIRS). The task performance showed a significantly increased reaction time (RT) in the negative valence independent of personality traits. The results of Oxy-Hb changes showed a significant interaction between personality traits and emotional valence. Further, the hemisphere-subgroup analysis revealed that the right DLPFC activity was significantly higher in the negative valence than in the neutral valence in the BIS group; the right DLPFC activity was also significantly higher in the BIS group than in the BAS group in the positive valence. There was no main effect or interaction in the left DLPFC activity. These findings suggest the importance of considering personality traits when examining the impact of emotional stimuli. Further studies with large sample sizes warranted to examine the influence emotional stimuli exert on working memory performance, considering the personality traits to better understand individual differences in emotional processing.

## Introduction

Cognitive control, a process that can inhibit automatic responses and support adaptive responses and goal-directed thought, can influence attention and memory (Dolcos and McCarthy, 2006; Dolcos et al., 2011; Inzlicht et al., 2015). Cognitive control can be associated with inhibition and promotion of emotional stimuli and be modulated by emotional salience. Some previous studies have reported that emotional salience changes the cognitive control load and indirectly affects attention and memory (Dolcos and McCarthy, 2006; Dolcos et al., 2011; Jasinska et al., 2012; Brosch et al., 2013). Also, cognitive control, related to accurate emotional perception and efficient control of the perceived emotion, plays an important role in selecting the appropriate response in emotional situations (Keil and Ihssen, 2004; Pessoa and Ungerleider, 2004; Inzlicht et al., 2015).

Previous research has shown two types of emotional processing: bottom-up and top-down (Norman and Bobrow, 1975). In bottom-up processing, emotion is implicitly triggered by stimuli regardless of the characteristics of the task, and the arising emotion affects cognitive function (Aoki et al., 2011). Subcortical areas including the amygdala are involved in this process (Hare et al., 2005; Phelps and LeDoux, 2005). Conversely, in top-down processing, the emotion is controlled by the dorsolateral prefrontal cortex (DLPFC; Erk et al., 2006; Waugh et al., 2010; Dixon et al., 2017). Previous neuroimaging evidence has indicated asymmetrical activation of the DLPFC in response to emotional stimuli; stimuli with negative emotional valence, i.e., that are intrinsically aversive, preferentially activate the right DLPFC and those with positive emotional valence, i.e., that are intrinsically attractive, preferentially activate the left DLPFC (Ortony et al., 1990; Tomarken et al., 1990; Wheeler et al., 1993; Solomon and Stone, 2002; Davidson, 2003; Russell, 2003; Balconi et al., 2017). These results suggest the importance of focusing on the interaction between prefrontal functional lateralization and stimulus emotional valence.

Personality is also reportedly related to emotion, leading to individual differences in emotional processing (Balconi and Mazza, 2010; Bendall et al., 2016; Balconi et al., 2017). Previous studies that investigated prefrontal activity during emotional stimulus presentation found that some participants had increased prefrontal activity in response to stimuli regardless of their emotional valence, whereas others showed an increase in a prefrontal activity dependent on stimulus emotional valence (Hoshi et al., 2011; Ozawa et al., 2014). One possible reason for this might be differences in emotional sensitivity, i.e., the degree of emotional responsiveness, among participants. Although there are numerous different types of personality tests, the Behavioral Inhibition/Behavioral Activation System (BIS/BAS) scale is often used in the field of emotional research based on a motivational model specialized in sensitivity to external stimuli. The BIS/BAS scale primarily measures the two general motivational systems that underlie behavior based on the reinforcement sensitivity theory of personality (Carver and White, 1994). The BIS regulates the motivation to retreat from aversive or unpleasant stimuli, and the BIS score is related to sensitivity to negative emotion. Conversely, the BAS regulates the appetitive motivation to approach desired stimuli, and the BAS score is related to sensitivity to positive emotion (Gray, 1970, 1982, 1987; Carver and White, 1994). The BIS and BAS largely relate to emotional processing and are interpreted as personality traits that are related to behavioral data and the background of physiology and biology based on brain function (Gray, 1982, 1994, 2001). Thus, it is assumed that personality traits related to the BIS/BAS may be among the factors responsible for the individual differences found in previous studies. Balconi et al. (2017) showed that the BIS score was positively correlated with the resting-state prefrontal activity of the right hemisphere using near-infrared spectroscopy (NIRS) and electroencephalography and that the BIS score was also related to the activity of the right hemisphere in response to negative stimuli. Balconi et al. (2017) also showed that the BAS score was positively correlated with the resting-state activity of the left hemisphere and was also related to the activity of the left hemisphere in response to positive stimuli. These results suggest a specific link between the BIS/BAS and brain activity in response to emotional stimuli.

Working memory tasks are frequently used as cognitive tasks to measure the impact of emotional stimuli (Van Dillen et al., 2009; Hart et al., 2010; Kopf et al., 2013; Ozawa et al., 2014). Previous studies have used the influence of task-irrelevant emotional stimuli, as distractors, on the performance of cognitive tasks as an index of the impact of emotional stimuli. Inhibition of task-irrelevant emotional information is known to recruit lateral prefrontal regions (Beauregard et al., 2001; Blair et al., 2007). Ozawa et al. (2014) used task performance on a 1-back task or 3-back task after presenting two consecutive emotional images of negative or neutral valence to measure the differences in the impact of emotional stimuli in each valence. Both working memory and emotional processing are closely linked to the DLPFC (Owen et al., 2005). When an emotional stimulus is presented immediately before the working memory task, the response to emotional stimuli works preferentially, reaching the limit of working memory capacity that can be used for task performance, leading to poor working memory performance (Dolcos and McCarthy, 2006; Brosch et al., 2013). To disentangle the interaction effect between emotional valence and personality traits on the activity of the DLPFC, the activity of the DLPFC was measured by NIRS while participants performed a dual 2-back task (Jaeggi et al., 2003). A dual *n*-back task is a working memory task that is simultaneously presented with auditory-verbal and visuospatial stimuli, and participants are required to respond to whether the current stimulus is the same with the one presented two times back. Previous studies have employed visual working memory tasks and have revealed a specific contribution of the left and right DLPFCs on verbal and spatial working memory, respectively (Smith and Jonides, 1999; Baddeley, 2003). Also, previous studies have reported that task performance after emotional stimulus presentation may change depending on task modality (Gray et al., 2002) and the task load (Jasinska et al., 2012; Kopf et al., 2013) and have also reported that a high level of task difficulty is required to extract changes in task performance. Therefore, laterality control and high difficulty task load are required to precisely measure the impact of emotional stimuli. By employing the dual 2-back task that utilizes auditory-verbal and visuospatial presentation and high difficulty task load, two types of working memory (auditory-verbal and visuospatial working memory) were targeted.

The purpose of this study was to clarify the influence of personality traits on the impact of emotional stimuli, focusing on *n*-back task performance and brain activity changes. In this study, the impact of emotional stimuli was evaluated through the changes in working memory task performance and DLPFC activity after the presentation of emotional stimuli in line with previous studies (Van Dillen et al., 2009; Hart et al., 2010; Kopf et al., 2013; Ozawa et al., 2014). The participants were presented with negative, neutral, or positive images just before the dual 2-back task. At the end of the experiment, the participants completed a questionnaire about the BIS/BAS. This procedure enabled examining the interaction effect of emotion and personality traits on task performance and DLPFC activity. Additionally, the laterality of DLPFC activity was targeted because it remains elusive whether the left and right DLPFCs show different activity patterns during this task. NIRS for neural-data collection was employed because it does not require specific environments that constrain participant movement, such as the scanner room required for functional magnetic resonance imaging, and enables researchers to collect brain activity data in a relatively ecologically valid setting (Tuscan et al., 2013).

The hypothesis for the task performance was 2-fold. The combination of specific emotional valence and personality traits that cause greater distraction in the dual *n*-back task and high brain activity was targeted based on the aforementioned previous reports. First, participants with high BIS score would have increased error rate (ER) and reaction time (RT) in the *n-back* task after being presented with stimuli with negative valence; second, participants with high BAS score would have increased ER and RT in the *n-back* task after being presented with stimuli with positive valence. Similarly, two hypotheses for DLPFC activity were developed; increased activity of the right DLPFC for negative stimuli would occur only for participants whose BIS score would be high, and increased activity of the left DLPFC for positive stimuli would occur only for participants whose BAS score would be high.

## Materials and Methods

### Participants

The required sample size for the present study was calculated using *a priori* power analysis using G power 3.1 (Faul et al., 2009) based on the effect size *f*. The interaction of working memory task performance between the group and emotional valence was considered the primary endpoint based on a previous report (Kopf et al., 2013), and the sample size for achieving a 0.95 statistical power level and given effect size *f* = 0.355 using a 2 × 3 mixed-design analysis of variance (ANOVA) was calculated. This resulted in a sample size *n* = 24. To be most conservative, 20% was added considering the possibility of drop-out, outliers, and variation in the number of participants between the groups and finally planned for a sample size *n* = 29. Regarding sex differences in response to emotional stimuli, a previous meta-analysis noted greater subjective and physiological responses to negative emotional stimuli in women relative to men (Stevens and Hamann, 2012). Therefore, this study only recruited male participants. Thirty right-handed healthy young men were finally included in this study (mean age, 21.6 ± 0.9 years; range, 20–24 years; mean years of education, 14.9 ± 0.7 years, range, 13–16 years). All participants achieved a score higher than 70 points on the Edinburgh Handedness Questionnaire Inventory (Oldfield, 1971) and had no history of neurological or psychiatric disorders. No participant reported a personal or family history of psychiatric or neurological diseases. To exclude the severe depressive status, both components of the State-Trait Anxiety Inventory (STAI; Spielberger et al., 1970) and the Beck Depression Inventory (BDI; Beck et al., 1996) were administered. No participant met the criteria for severe depressive status. This study protocol was approved by the Ethics Committee of the Health Sciences University of Hokkaido (Approval number: 18R075067), and all experiments were performed following the latest version of the Declaration of Helsinki. All participants provided written informed consent before the experiment.

### Experimental Design

All participants were classified into two groups, a BIS and a BAS group, based on their average score on the BIS and BAS scales. Participants in both groups performed the dual *n*-back task after the presentation of emotional stimuli and their brain activity during the task was measured using NIRS. After performing the task, the participants were required to evaluate the emotional valence and arousal ratings of all emotional images that were presented immediately before the task and complete the BIS/BAS scale. These data were statistically analyzed to identify the effects of personality traits on the three emotional valences. For dual-*n* back performance, the ER (%) and RT (ms) of the dual *n-back* task after the presentation of the emotional stimuli were set as dependent variables and group (BIS or BAS) and emotional valence (negative, neutral, or positive) as the between- and within-subjects factors, respectively, were set as independent variables. To identify brain activities during the task, change in oxygenated hemoglobin (Oxy-Hb) concentration ([m(mol/l)*mm]) in the DLPFC was set as the dependent variable and group (BIS or BAS), as a between-subjects factor, and emotional valence (negative or neutral or positive) and hemisphere (left or right), as the within-subject factors, were set as independent variables.

### Personality Trait Status

The BIS/BAS scale, Japanese version (Takahashi et al., 2007) was used as a personality trait scale; it consists of seven BIS (score range, 7–28) and 13 BAS (score range, 13–52) items for a total of 20 items (total score range, 20–80). As the BIS/BAS subscales differ in the number of included items, the total score cannot be effectively used to determine which participants tended toward BI or BA. Therefore, all participants were classified into two groups based on their average score on the BIS and BAS subscales. The higher BIS-score group (BIS group) consisted of 11 participants (mean age, 21.0 ± 0.9 years; years of education, 14.7 ± 0.8; Edinburgh Handedness Questionnaire Inventory score, 92.6 ± 10.3; trait anxiety score, 49.6 ± 8.6; state anxiety score, 42.0 ± 7.1; BDI score, 10.8 ± 7.0, BIS score 21.9 ± 2.3, BAS score 36.8 ± 4.6), and the higher BAS-score group (BAS group) consisted of 19 participants (mean age, 21.9 ± 0.8 years; years of education, 15.0 ± 0.6; Edinburgh Handedness Questionnaire Inventory score, 92.0 ± 7.9; trait anxiety score, 42.6 ± 7.3; state anxiety score, 38.2 ± 6.4; BDI score, 5.2 ± 4.4, BIS score 16.7 ± 3.9, BAS score 42.1 ± 5.4).

### Emotional Stimuli

Emotional stimuli were selected from the International Affective Picture System (IAPS; Lang et al., 2008), which is frequently used in broad research areas (e.g., Kensinger and Corkin, 2003; Dolcos and McCarthy, 2006; Ito et al., 2011; Ozawa et al., 2014; Balconi et al., 2017). In the IAPS, emotional valence and the arousal level of the emotional images are scored on a 9-point rating scale. The IAPS is a widely used tool not influenced by cultural and customary factors. While individual differences in sensitivity for emotional stimuli according to one’s experiences and nature and intensity of emotion has also been reported (Bloise and Johnson, 2007). Therefore, it was considered prudent to ensure that the values of the IAPS are consistent in the population in this study by conducting a preliminary experiment as did previous studies (Costa et al., 2014; Balconi et al., 2017). A preliminary experiment was conducted to extract appropriate stimuli for use in this study from several IAPS images.

Thirty right-handed healthy young men who did not participate in the main study were recruited for the preliminary experiment (mean age, 22.2 ± 1.2 years; range, 21–25 years). All participants provided written informed consent before the experiment.

For the experimental procedure, three images served as controls for the scaling criteria and five each for positive (mean valence, 8.0 ± 0.2, mean arousal, 5.1 ± 0.7), neutral (mean valence, 5.1 ± 0.1, mean arousal, 3.1 ± 0.2), and negative (mean valence, 1.7 ± 0.3, mean arousal, 6.5 ± 0.6) valence based on the established average emotional valence rating and their *SD* value were extracted; in total, 15 images were extracted from 1194 included in the IAPS (Supplementary Table 1). Each of these images was randomly presented on a computer screen, and then, the valence and arousal ratings of all images were evaluated by the participants using the Self-Assessment Manikin Scale on a 9-point Likert scale (Bradley and Lang, 1994). Two images with the lowest average emotional valence rating (lower than 2.0), two with an average rating close to the median (approximately, 5.0), and two with the highest average rating (higher than 7.5) were utilized as the negative, neutral, and positive stimuli, respectively. The Kruskal–Wallis test and the Steel–Dwass test for multiple comparisons were performed to confirm the differences in valence and arousal ratings among these negative, neutral, and positive images. Additionally, a one-sample *t*-test for the typical neutral emotional valence rating (5.0) was performed to ensure the validity of the valence classification in these images.

The average emotional valence rating was 1.6 ± 0.8 in negative, 5.0 ± 0.3 in neutral, and 7.8 ± 0.8 in positive images. Moreover, the statistical analysis revealed significant differences among the three emotional valences (Kruskal–Wallis test: *H*_(2)_ = 156.940, *p* < 0.001, *post hoc* tests: Negative < Neutral < Positive; all, *p* < 0.01, $\eta_{\text{p}}^{2}$ = 0.875). In addition, the average arousal rating in each emotional valence was 7.2 ± 2.3 in negative, 2.9 ± 1.6 in neutral, 6.0 ± 2.1 in positive images (Kruskal–Wallis test: *H*_(2)_ = 73.605, *p* < 0.001, *post hoc* tests: Negative > Positive > Neutral; all, *p* < 0.05, $\eta_{\text{p}}^{2}$ = 0.405). The six emotional pictures selected based on the results of this preliminary experiment were used in the main part of the study.

To verify that the participants perceived the emotional image stimuli, the skin conductance response (SCR) was measured simultaneously and continuously during the NIRS measurement using the UFI Model 2701 BioDerm™ Skin Conductance Meter (UFI, Morro Bay, CA, USA).

### Dual *n*-Back Task

**Figure 1:**
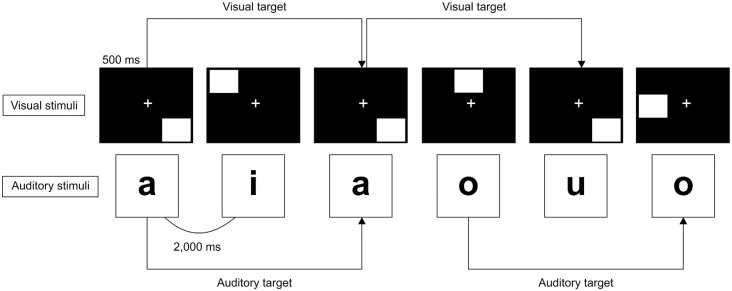
Dual 2-back task. The auditory-verbal and visuospatial stimuli are presented simultaneously and consecutively. The participants are required to answer whether the current stimulus is the same as the stimulus presented two times back as quickly and accurately as possible for each stimulus. Each stimulus was presented for 500 ms, and the interstimulus interval was 2,000 ms.

The *n*-back task is a working memory task that presents a series of stimuli in order. The participant needs to answer whether the currently presented stimulus is the same as the stimulus presented *n* times back. The level of task difficulty is modulated by the loading factor *n*. The dual *n-back* task consisted of simultaneously presented auditory-verbal and visuospatial *n-back* tasks and required participants to memorize the sequences of items presented (Jaeggi et al., 2003; Buschkuehl et al., 2007). The dual *n-back* task was used as the working memory task. The series of auditory-verbal and visuospatial stimuli were simultaneously presented on the sound speaker and computer monitor using the DMDX display software (University of Arizona, Tuscon, AZ, USA; Foster and Foster, 2003), and a randomly selected white square in eight different locations except for the central position within an invisible grid in a 3 × 3 square was visually presented on the screen. Moreover, a randomly selected Japanese kana phonetic character (e.g., “a,” “i,” “u,” “e,” “o”) was orally presented at the same rate of 2,000 ms per stimulus. Each of the auditory-verbal and visuospatial stimuli was presented for 500 ms. The tasks consisted of six blocks (two blocks for each valence) and each block consisted of 16 stimuli (25% both auditory-verbal and visuospatial stimuli match, 50% either of these matches, 25% neither of these matches) with a total duration of 40.0 s. In the dual 2-back task (Figure 1), the participants were required to determine as quickly and accurately as possible whether the current stimulus matched the one that was presented two times back. The participants were required to respond whether the current stimuli matched the stimuli presented two times back or not as follows: pressing the cursor key left (←) when either of the auditory-verbal and visuospatial stimuli matched, pressing the cursor key right (→) when both of these matched, and not pressing any key when neither of these matched. The ER and RT of the dual 2-back task were calculated and considered the task performance.

### Neuroimaging System

The changes in Oxy-Hb concentration were measured with a multi-channel functional NIRS (fNIRS) optical topography system (LABNIRS, Shimadzu Corporation Kyoto, Japan) with three wavelengths of near-infrared light: (780, 805, and 830 nm). The fNIRS probes consisted of 16 illuminating and 14 detecting probes arranged alternately at an inter-probe distance of 3 cm, resulting in 44 channels according to the international 10-20 placement system. The sampling rate was 6.17 Hz.

**Figure 2:**
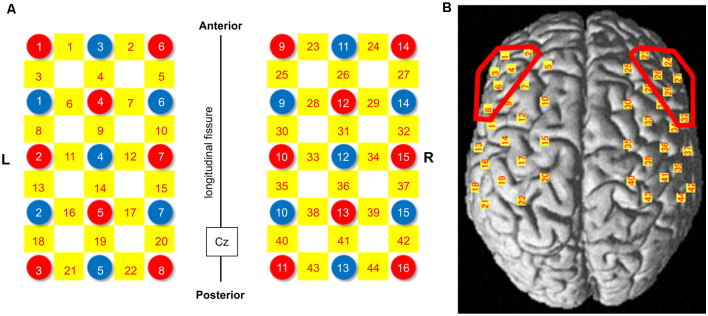
NIRS probe arrangement (Left 3 × 5, Right 3 × 5). **(A)** Illuminators and detectors are shown as red and blue circles, respectively, and channels are shown as yellow squares. The Cz was defined according to the international 10-20 placement system. **(B)** The channel positions are shown on the cortical surface. Red-colored circles show the dorsolateral prefrontal cortex (DLPFC) area. NIRS: near-infrared spectroscopy.

These probes were placed over a broad cortical area including the DLPFC, which was set as the region of interest (ROI), and the fNIRS probes were arranged in a 3 × 5 square on the left and a 3 × 5 square on the right (Figure 2). The fNIRS optode and reference positions (Cz, Nz, Iz, AL, and AR) were digitized using a three-dimensional digitizer (FASTRAK; Polhemus, Colchester, VT, USA). The coordinate data were registered into the Montreal Neurological Institute coordinates using the “coordinate-based system” function in NIRS_SPM (Supplementary Table 2). The anatomical location of each channel was determined according to the Talairach Daemon (Talairach and Tournoux, 1988; Lancaster et al., 1997). The anatomical labeling (Brodmann areas, Talairach Daemon), which was averaged in all participants, is listed for each channel in Table 1. Two participants were excluded from this analysis because of missing data due to device malfunction. Channels 1, 2, 3, 4, 6, and 8 were included for the left DLPFC, and channels 23, 24, 26, 27, 29, and 32 were included for the right DLPFC. All channels included in these ROIs exceeded 60% of the estimated probability in individual-level registration, indicating the validity of this procedure in ensuring the accuracy of spatial registration.

**Table 1:** Anatomical labeling of near-infrared spectroscopy (NIRS) channel positions.

Anatomical labeling		Average overlap probability (%)		Channel number	
Talairach Daemon	Brodmann area		Left	Right
**Dorsolateral prefrontal cortex**	**9, 46**	**72.0 (1.8)**	**1, 2, 3, 4, 6, 8**	**23, 24, 26, 27, 29, 32**
Includes Frontal eye fields	8	85.4 (2.2)	5, 7, 9	25, 28, 31
Frontopolar area	10	26.5 (2.0)	1, 2	23, 24
Primary Motor Cortex	4	44.5 (0.3)	20	40
Pre-Motor and Supplementary Motor Cortex	6	90.2 (1.5)	10, 11, 12, 13, 14, 15, 17	30, 33, 34, 35, 36, 37, 38
Primary Somatosensory Cortex	1, 2, 3	68.0 (2.4)	16, 19, 22	39, 41, 43
Somatosensory Association Cortex	5, 7	36.2 (1.4)	22	43
Supramarginal gyrus part of Wernicke’s area	40	78.4 (3.0)	18, 21	42, 44
Pars triangularis Broca’s area	45	14.5 (1.1)	3, 8	27, 32

*The values in parenthesis show the standard error. NIRS: near-infrared spectroscopy. Bold values show the information about the region of interest*.

In this study, the baseline period comprised 24–10 s before task onset, and the average Oxy-Hb value of the baseline period was set to zero. A bandpass filter was then applied between 0.01 and 0.30 s. To avoid NIRS pathlength issues, the changes in Oxy-Hb concentration during the task were calculated as the difference from the baseline value (Hoshi, 2003).

### Experimental Procedure

The laboratory room was isolated, and the lighting in the room was maintained stable to avoid emotional input derived from the surrounding environment. Participants sat on a chair in front of a 27-inch computer monitor and gazed at a fixation point on the screen to reduce eye and head movements. The visual angle from the center of the fixation cross to the edge of the monitor was set at 7°, and a sound system speaker was installed in front of the participants. The participants also wore the NIRS head cap and skin conductance electrode on their left finger and were instructed to avoid head and body motion and deep breathing during the NIRS measurements.

**Figure 3:**
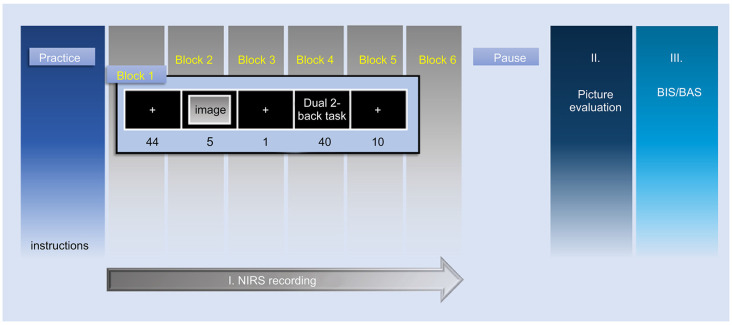
Representation of the time course of the experimental protocol. This study consisted of the following three phases; I: NIRS-recording phase, II: Image-evaluation phase, III: BIS/BAS-scale phase. Phase I shows the NIRS measurement by block design. NIRS measurement was performed in six blocks according to the number of emotional images presented to each participant (two images for every emotional valence condition, six images in total). NIRS, near-infrared spectroscopy; BIS, behavioral inhibition system; BAS, behavioral activation system.

The experimental procedure, after the first practice phase, consisted of three phases; I: NIRS-recording phase (picture presentation and n-bac task phase), II: Image-evaluation phase, and III: BIS/BAS-scale phase (Figure 3). At the first practice phase, the participants received a demonstration of the task and performed the dual 2-back task in a short period. A block design was used in the NIRS measurement; the protocol consisted of six blocks according to the number of the presented emotional images, i.e., two images for each emotional valence condition, six images in total. The presentation order of these images and the stimuli in the dual 2-back task were randomized in each participant to avoid order effects.

In the first resting period of phase I, a white cross fixation was displayed at the center of the screen for 49.0 s. The participants were instructed to gaze at the white cross fixation. Subsequently, the emotional image was presented on the computer screen for 5.0 s. After a 1,000 ms fixation, the 2-back task was conducted for 40.0 s. fNIRS measurements were acquired through the whole of phase I: NIRS-recording phase. In phase II, the participants evaluated the emotional valence and arousal ratings of all emotional images with the evaluation methods used in the preliminary experiment.

### Statistical Analysis

To examine differences in valence and arousal levels among the three emotional valence conditions, the Kruskal–Wallis test was performed, followed by the Steel–Dwass test for multiple comparisons. The SCR was calculated from the waveform data measured by the Skin Conductance Meter, and the peak SCR values during the rest period and 3.0 s after image presentation were extracted. A paired *t*-test was performed to compare the differences between them.

For dual-textitn back performance, two 2 × 3 mixed-design ANOVAs with a group (BIS or BAS) and stimulus valence (negative, neutral, or positive) as the between- and within-subjects’ factors, respectively, were performed separately for ER and RT. To identify ROI activities during the task, changes in Oxy-Hb concentration in the ROIs were analyzed using a 2 × 3 × 2 mixed-design ANOVA with group (BIS or BAS) as a between-subjects factor and emotional valence (negative or neutral or positive) and hemisphere (left or right) as the within-subject factors. Furthermore, to confirm the relationship among the three factors [personality traits (total BIS and BAS scores), task performance (ER and RT) on the dual 2-back task, and DLPFC activity (ΔOxy-Hb in the left and right DLPFCs)] in each of the three emotional valence conditions, the Spearman’s rank correlation coefficient was calculated. All statistical analyses were performed using SPSS 23.0 (IBM-SPSS Inc., Armonk, NY, USA), and the statistical significance level was set to 0.05.

## Results

### Demographic Data

No significant differences in age, years of education, Handedness Questionnaire Inventory score, and state anxiety score were observed between the two groups (all, *p* > 0.05). However, significantly higher trait anxiety score (BIS group mean, 49.6 ± 8.6, BAS group mean, 42.6 ± 7.3, *t*_(28)_ = −2.384, *p* = 0.024, *d* = 0.940) and BDI scores (BIS group mean, 10.8 ± 7.0, BAS group mean, 5.2 ± 4.4, *t*_(28)_ = −2.704, *p* = 0.012, *d* = 1.060) were shown in the BIS than in the BAS group.

### Verification of Emotional Image Stimuli

The average emotional valence rating in each emotional valence condition was 2.1 ± 1.1 in negative, 5.2 ± 0.6 in neutral, and 7.2 ± 1.0 in positive images, and the statistical analysis revealed significant differences among the three emotional valence conditions (Kruskal–Wallis test: *H*_(2)_ = 148.368, *p* < 0.001, *post hoc* tests: Negative < Neutral < Positive; all, *p* < 0.01, $\eta_{\text{p}}^{2}$ = 0.827). In addition, the average arousal rating in each emotional valence condition was 6.9 ± 1.6 in negative, 3.4 ± 2.0 in neutral, and 5.3 ± 2.3 in positive images (Kruskal–Wallis test: *H*_(2)_ = 70.694, *p* < 0.001, *post hoc* tests: Negative > Positive > Neutral; all, *p* < 0.01, $\eta_{\text{p}}^{2}$ = 0.388). These results were very similar to those of the preliminary experiment (Supplementary Figure 1).

In the SCR analysis, to ensure the perceptual input of emotional image stimuli, the average of the SCR during the image presentation was significantly higher than those during rest in all emotional valence conditions (all, *p* < 0.01; Supplementary Figure 2). These results could mean that the participants perceived the emotional image stimuli.

### Behavioral Results

**Figure 4:**
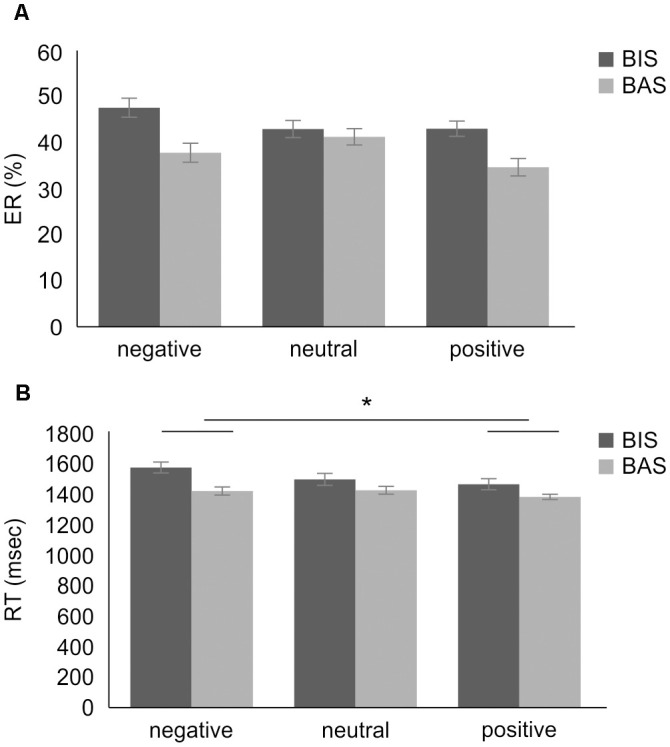
The results of the two-way mixed-design ANOVA for dual 2-back task performance. **(A)** The results of the two-way mixed-design ANOVA of ER. There was no significant main effect and interaction. **(B)** The results of the two-way mixed-design ANOVA of the RT. There was a significant main effect of emotional valence, but no interaction. The *post hoc* test showed significantly increased RT in the negative compared with the positive valence condition. *p < 0.01. Error bars indicate the standard error. ANOVA, analysis of variance; BIS, behavioral inhibition system; BAS, behavioral activation system; ER, error rate; RT, reaction time.

The ER’s 2 × 3 mixed-design ANOVA revealed no significant main effect of group (*F*_(1,28)_ = 0.683, mean square error (MSE) = 0.095, *p* = 0.416, $\eta_{\text{p}}^{2}$ = 0.024) and emotional valence (*F*_(2,56)_ = 1.461, *MSE* = 0.013, *p* = 0.241, $\eta_{\text{p}}^{2}$ = 0.050). Furthermore, no interaction effect was observed (*F*_(2,56)_ = 1.392, *MSE* = 0.012, *p* = 0.257, $\eta_{\text{p}}^{2}$ = 0.047). In addition, planned contrasts were performed for the interaction effects; as these were planned *a priori*, there was no requirement for the omnibus *F* value to reach significance. The planned contrasts revealed no significant between-group difference in ER in the negative valence condition (BIS group mean ER, 0.479 ± 0.164, BAS group mean ER, 0.378 ± 0.248, *t*_(27.335)_ = −1.332, *p* = 0.194, *d* = 0.470), and in the positive valence condition (BIS group mean ER, 0.423 ± 0.194, BAS group mean ER, 0.348 ± 0.242, *t*_(28)_ = −0.954, *p* = 0.348, *d* = 0.370; Figure 4A).

Conversely, the RT’s 2 × 3 mixed-design ANOVA revealed a significant main effect of emotional valence (*F*_(2,56)_ = 4.321, *MSE* = 45976.993, *p* = 0.018, $\eta_{\text{p}}^{2}$ = 0.134; Figure 4B). The *post hoc* test showed significant RT increases in the stimuli with negative valence (mean RT, 1470.4 ± 237.3 ms) compared to the stimuli with positive valence condition (mean RT, 1397.9 ± 251.5 ms, *t*_(29)_ = 2.646, corrected *p* = 0.040, *d* = 0.297), but no significant RT changes in the stimuli with negative compared to neutral valence condition (mean RT, 1445.3 ± 247.7 ms, *t*_(29)_ = 0.896, corrected *p* = 1.000, *d* = 0.104), and in the stimuli with neutral compared to positive valence condition (*t*_(29)_ = 1.962, corrected *p* = 0.178, *d* = −0.191). No significant main effect of group (*F*_(1,28)_ = 1.413, *MSE* = 222542.203, *p* = 0.245, $\eta_{\text{p}}^{2}$ = 0.048) and interaction effect (*F*_(2,56)_ = 0.902, *MSE* = 9595.864, *p* = 0.412, $\eta_{\text{p}}^{2}$ = 0.031) was observed in the 2 × 3 RT’s mixed-design ANOVA (Figure 4B). The planned contrasts revealed no significant between-group difference in RT in the negative valence condition (BIS group mean RT, 1562.9 ± 119.7 ms, BAS group mean RT, 1416.9 ± 273.0 ms, *t*_(26.649)_ = −2.020, *p* = 0.054, *d* = 0.660), and in the positive valence condition (BIS group mean RT, 1450.7 ± 215.8 ms, BAS group mean RT, 1367.3 ± 270.9 ms, *t*_(28)_ = −0.871, *p* = 0.391, *d* = 0.340).

### DLPFC Activity Analysis Results

In the ROI activity analysis, the data satisfying the Oxy-Hb concentrations of the bilateral DLPFC at rest within ± 2 SD was used. Two cases exceeded this range and were excluded. Therefore, 10 and 18 participants were included in the BIS and BAS groups, respectively, for a total of 28 participants included in this analysis.

The 2 × 3 × 2 mixed-design ANOVA for the Oxy-Hb concentration changes in the DLPFC revealed no significant main effect of group (*F*_(1,26)_ = 0.597, *MSE* = 0.000017, *p* = 0.447, $\eta_{\text{p}}^{2}$ = 0.022), emotional valence (*F*_(2,52)_ = 0.699, *MSE* = 0.000005, *p* = 0.502, $\eta_{\text{p}}^{2}$ = 0.026), and hemisphere (*F*_(1,26)_ = 0.643, *MSE* = 0.000008, *p* = 0.430, $\eta_{\text{p}}^{2}$ = 0.024). Conversely, a significant interaction effect of group × emotional valence (*F*_(2,52)_ = 3.492, *MSE* = 0.000026, *p* = 0.038, $\eta_{\text{p}}^{2}$ = 0.118) was observed. The simple main effect of group was not significant in the stimuli with negative valence condition (BIS group mean, 0.0022 ± 0.0029 [m(mol/l)*mm], BAS group mean, 0.0014 ± 0.0032 [m(mol/l)*mm], *t*_(26)_ = −0.669, *p* = 0.509, *d* = 0.270), in the stimuli with neutral valence condition (BIS group mean, 0.0008 ± 0.0026 [m(mol/l)*mm], BAS group mean, 0.0016 ± 0.0020 [m(mol/l)*mm], *t*_(26)_ = 0.965, *p* = 0.343, *d* = 0.400), or in the stimuli with positive valence condition (BIS group mean, 0.0028 ± 0.0020 [m(mol/l)*mm], BAS group mean, 0.0008 ± 0.0029 [m(mol/l)*mm], *t*_(26)_ = −1.940, *p* = 0.063, *d* = 0.790). Similarly, one-way ANOVA showed that the simple main effect of emotional valence was not significant in the BIS (*F*_(2,18)_ = 3.066, *MSE* = 0.000010, *p* = 0.071, $\eta_{\text{p}}^{2}$ = 0.254) and BAS groups (*F*_(2,34)_ = 0.915, *MSE* = 0.000004, *p* = 0.410, $\eta_{\text{p}}^{2}$ = 0.051).

**Figure 5:**
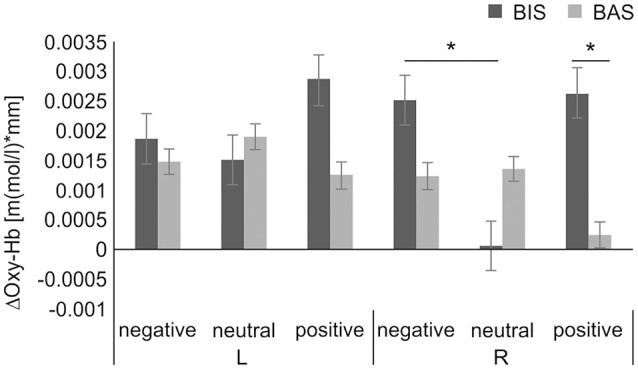
ΔOxy-Hb concentration in the DLPFC during the dual 2-back task. There was a significant group × emotional valence interaction. The *post hoc* test showed significantly higher ΔOxy-Hb concentration in the right DLPFC in the negative compared with the neutral valence condition in the BIS group and significantly higher ΔOxy-Hb concentration in the right DLPFC in the BIS compared with BAS group in the positive valence condition. *p < 0.01. Error bars indicate the standard error. DLPFC, dorsolateral prefrontal cortex; ΔOxy-Hb, difference in oxygenated hemoglobin; BIS, behavioral inhibition system; BAS, behavioral activation system.

In addition, a further subgroup analysis of each hemisphere, based on our hypothesis, was performed to analyze the effect of personality on the interaction of group × emotional valence. The 2 × 3 mixed-design ANOVA of the Oxy-Hb concentration changes in the DLPFC with group (BIS or BAS), as a between-subjects factor, and emotional valence (negative or neutral or positive) as the within-subjects factor, revealed no significance in the main effects of group in the left (*F*_(1,26)_ = 0.213, *MSE* = 0.000007, *p* = 0.648, $\eta_{\text{p}}^{2}$ = 0.008) and right DLPFC (*F*_(1,26)_ = 0.768, *MSE* = 0.000012, *p* = 0.389, $\eta_{\text{p}}^{2}$ = 0.029), or the emotional valence in the left (*F*_(2,52)_ = 0.155, *MSE* = 0.000001, *p* = 0.857, $\eta_{\text{p}}^{2}$ = 0.033) and right DLPFC (*F*_(2,52)_ = 1.964, *MSE* = 0.000009, *p* = 0.151, $\eta_{\text{p}}^{2}$ = 0.070). Conversely, a significant interaction effect of group × emotional valence in the right DLPFC (*F*_(2,52)_ = 5.055, *MSE* = 0.000023, *p* = 0.010, $\eta_{\text{p}}^{2}$ = 0.163) was observed. The simple main effect of group was significant in the stimuli with positive valence condition (*F*_(1,26)_ = 9.608, *MSE* = 0.000036, *p* = 0.005, $\eta_{\text{p}}^{2}$ = 0.270), but not significant in the stimuli with negative valence condition (*F*_(1,26)_ = 0.782, *MSE* = 0.000010, *p* = 0.384, $\eta_{\text{p}}^{2}$ = 0.029), in the stimuli with neutral valence condition (*F*_(1,26)_ = 1.462, *MSE* = 0.000011, *p* = 0.237, $\eta_{\text{p}}^{2}$ = 0.053). Furthermore, the simple main effect of emotional valence was significant in the BIS group (*F*_(2,52)_ = 4.655, *MSE* = 0.000021, *p* = 0.014, $\eta_{\text{p}}^{2}$ = 0.152), but not significant in the BAS group (*F*_(2,52)_ = 1.448, *MSE* = 0.000006, *p* = 0.244, $\eta_{\text{p}}^{2}$ = 0.053). The *post hoc* test revealed significantly higher right DLPFC Oxy-Hb concentration changes in the stimuli with negative valence (mean, 0.0025 ± 0.0035 [m(mol/l)*mm]) than in the neutral valence condition in the BIS group (mean, 0.0001 ± 0.0034 [m(mol/l)*mm], *t*_(9)_ = 3.253, corrected *p* = 0.030, *d* = 0.709), but no significant right DLPFC Oxy-Hb concentration changes in the BIS group in the stimuli with negative compared to positive valence condition (mean, 0.0026 ± 0.0015 [m(mol/l)*mm], *t*_(9)_ = −0.126, corrected *p* = 1.000, *d* = −0.049), and in the stimuli with neutral compared to positive valence condition (*t*_(9)_ = −2.400, corrected *p* = 0.120, *d* = −0.976). Additionally, the *post hoc* test revealed significantly higher Oxy-Hb concentration changes in the right DLPFC in the BIS (mean, 0.0026 ± 0.0015 [m(mol/l)*mm]) than in the BAS group in the stimuli with positive valence condition (mean, 0.0003 ± 0.0021 [m(mol/l)*mm], *t*_(26)_ = −3.100, *p* = 0.005, *d* = 0.690), but no significant right DLPFC Oxy-Hb concentration changes between these groups in the stimuli with negative valence condition (BIS group mean, 0.0025 ± 0.0035 [m(mol/l)*mm], BAS group mean, 0.0012 ± 0.0037 [m(mol/l)*mm], *t*_(26)_ = −0.885, *p* = 0.384, *d* = 0.360). There was no interaction effect of group × emotional valence in the left DLPFC (*F*_(2,52)_ = 0.883, *MSE* = 0.000007, *p* = 0.419, $\eta_{\text{p}}^{2}$ = 0.033). In addition, the planned contrasts revealed no significance in the left DLPFC Oxy-Hb concentration changes between these groups in the stimuli with positive valence condition (BIS group mean, 0.0029 ± 0.0033 [m(mol/l)*mm], BAS group mean, 0.0013 ± 0.0048 [m(mol/l)*mm], *t*_(26)_ = −0.939, *p* = 0.356, *d* = 0.380), and in the stimuli with negative valence condition (BIS group mean, 0.0019 ± 0.0036 [m(mol/l)*mm], BAS group mean, 0.0015 ± 0.0040 [m(mol/l)*mm], *t*_(26)_ = −0.245, *p* = 0.808, *d* = 0.100; Figure 5).

There was no interaction effect of group × hemisphere (*F*_(1,26)_ = 0.048, *MSE* = 0.000001, *p* = 0.829, $\eta_{\text{p}}^{2}$ = 0.002) or of emotional valence × hemisphere (*F*_(2,52)_ = 1.083, *MSE* = 0.000005, *p* = 0.346, $\eta_{\text{p}}^{2}$ = 0.040). Furthermore, there was no significant interaction effect of group × emotional valence × hemisphere (*F*_(2,52)_ = 0.735, *MSE* = 0.000003, *p* = 0.484, $\eta_{\text{p}}^{2}$ = 0.027).

### Correlation Analysis

There was no significant correlation between task performance (ER and RT) and DLPFC activity (ΔOxy-Hb concentration changes in the left and right DLPFCs) for any emotional valence condition (all, *p* > 0.05; Supplementary Table 3).

## Discussion

The influence of personality traits on the impact of emotional stimuli was examined in this study focusing on *n*-back task performance and brain activity changes. A 2-fold hypothesis that task performance would decrease after the presentation of negative stimuli in the BIS group and positive stimuli in the BAS group was established. The results showed significantly increased RT in the dual 2-back task in the stimuli with negative valence condition. However, against the hypothesis, no significantly increased RT could be found in the dual *n-back* task after the presentation of negative stimuli in the BIS group and positive stimuli in the BAS group. Also, against the hypothesis, no significantly increased ER could be found in the dual *n-back* task after the presentation of stimuli with negative valence in the BIS group and with positive valence in the BAS group. Moreover, two hypotheses were developed, i.e., that, in the BIS group, the activity would increase after presentation of negative stimuli in the right DLPFC, and in the BAS group, the activity would increase after presentation of positive stimuli in the left DLPFC. The results showed that the right DLPFC activity was increased after the presentation of negative stimuli in the BIS group suggesting the importance of considering personality traits when examining the effect of individual differences on the impact of emotional stimuli. However, left DLPFC activity did not increase after the presentation of positive stimuli in the BAS group. Also, significantly increased brain activity was observed in response to stimuli with positive valence in the BIS group, which is a result not anticipated by the hypothesis.

Regarding the task performance results, an increased RT was only observed in the stimuli with negative valence condition. Some previous studies have reported that negative stimuli would induce strong cognitive task performance degradation regardless of personality trait differences. A previous study, which used the IAPS (Ozawa et al., 2014), reported that RT tended to increase in a verbal *n*-back task in negative compared to neutral images. Moreover, Kopf et al. (2013) also reported that verbal *n-back* task performance decreased after the presentation of negative stimuli depending on task difficulty. The present results are similar to those of these studies in terms of RT increases in the stimuli with negative valence condition. One of the reasons for decreasing task performance after the presentation of negative stimuli may be the more sustained effect of negative stimuli over time (Dolcos and McCarthy, 2006; Waugh et al., 2010, 2014). Particularly, it could be considered that negative stimuli operate as a long-lasting distractor in the impact of emotional stimuli (Jasinska et al., 2012). Conversely, differences in the gross BDI score and trait anxiety score between these two groups could have affected the result of increased RT in the stimuli with negative valence condition because the gross BDI score and trait anxiety score were significantly higher in the BIS than in the BAS group. Numerous previous studies have reported that patients with depression showed decreased working memory performance (Semkovska et al., 2019). However, the present findings showed no significant difference in dual *n-back* task performance in the neutral condition between these two groups. Therefore, the differences within the normal range in the BDI score, which assesses the depressive state, may suggest that it did not largely affect working memory performance in this study.

Furthermore, similar results to those of previous studies were obtained in terms that an emotional stimulus effect was observed in RT but not in ER (Kensinger and Corkin, 2003; Dresler et al., 2009; Hart et al., 2010). Dresler et al. (2009) reported that RT in the Stroop task was affected by emotional stimulus arousal instead of emotional valence. Additionally, Kensinger and Corkin (2003) noted that RT is sensitive to emotional interference effects; the efficiency of information processing speed in a working memory task is affected by emotion stimuli. Moreover, Ozawa et al. (2014) reported that RT would increase in parallel with task difficulty. Many previous studies have used low difficulty tasks with lower than 10% ER (Gray, 2001; Kopf et al., 2013; Ozawa et al., 2014; Ozawa and Hiraki, 2017; Ozawa et al., 2019). However, a high difficulty task with approximately 40–50% ER was used in this study. Consequently, the fact that an emotional stimulus effect was only observed for RT may indicate that RT is influenced by task difficulty and emotional stimulus arousal. However, this study focused on emotional valence, not emotional arousal, and is limited in that the involvement of arousal was not addressed because the control of negative and positive arousal levels was not considered.

The decrease in task performance was not observed in the stimuli with negative valence condition in the BIS and positive valence condition in the BAS group. These results did not support the hypothesis. Several reasons for this result could be considered. First, a dual 2-back task was employed, composed of auditory-verbal and visuospatial stimuli because the purpose of this study was to examine hemisphere-specific activity in the DLPFC and clarify the effect of personality traits on the impact of emotional stimuli; hemisphere-specific activity in the DLPFC would have depended on the type of task modality (i.e., visuospatial or verbal). Especially, both the verbal *n*-back task and positive stimuli would have increased left hemisphere activity. Thus, competitive DLPFC activity can occur in the left hemisphere and it would contribute to the degradation of task performance observed in previous studies (Kopf et al., 2013; Ozawa et al., 2014). Moreover, the results would be expected to be more robust in the BAS group because higher left hemisphere activity is expected in BAS groups. Another possible reason for task performance decrease may involve differences in task difficulty (Jasinska et al., 2012). A dual 2-back task of high difficulty was used in this study. RT, similar to ER, depends on task difficulty in the *n-back* task. Previous studies using *n-back* tasks have shown that the average RT in all emotional valence conditions was lower than 1,000 ms, but the average RT in this study was higher than 1,500 ms. Kopf et al. (2013) reported that there were no significant differences in task performance with stimuli with positive valence presentation at high cognitive loads, but there were significant decreases in task performance with stimuli with negative valence presentation alone. Furthermore, the longer task duration influenced task performance. In this study, a task duration of 40 s was employed, whereas previous studies adopted task durations of 16–26 s. This longer task duration would attenuate the effect of positive as opposed to negative stimuli, sustaining the effect for a longer time. Considering these reasons, differences in task performance caused by different emotional valence would depend on the nature of the task including the engaged sensory modality, task difficulty, and task duration. Also, Gray (2001) considered the role of personality traits in task performance and reported that spatial working memory task performance would decrease after the presentation of negative compared to positive images. Besides, Gray (2001) also reported decreased performance after the presentation of negative images in a BIS group compared with a BAS group and suggested that personality traits may have affected the decreased task performance triggered by emotional stimuli. In this study, the decrease in task performance after the presentation of negative stimuli did not reach statistical significance in the BIS group, but the effect size of planned contrast (negative vs. positive) showed a moderate effect size (Cohen’s *d* = 0.660). This result may suggest that larger sample size could resolve the discrepancies between this and the previous study.

In the NIRS measurement, anatomical labeling with spatial registration was conducted to ensure the spatial accuracy of the DLPFC, which was set as an ROI. This methodological process is considered to be novel because previous studies used a channel level (Ozawa et al., 2014) and did not examine the laterality of DLPFC activity (Kopf et al., 2013). Regarding brain activity during the task, the hypothesis was that high brain activity would be found because of information processing competition for combinations of specific emotional valence and personality traits. The results showed significantly increased right DLPFC activity following the presentation of negative compared to neutral stimuli in the BIS group (Figure 5). Previous studies have reported that Oxy-Hb concentration strongly increased in the right DLPFC during a task after the presentation of negative stimuli (Kopf et al., 2013; Compare et al., 2016) regardless of personality traits. The present results support the hypothesis that negative stimulus presentation contributes toward increased right DLPFC activity. Based on the relationship between personality traits and DLPFC lateralization, the BIS group might have shown more robust increases of right DLPFC activity in this study compared to the findings of these previous studies.

Additionally, as a result not covered by the hypothesis, increased right DLPFC activation was observed in the stimuli with positive valence condition in the BIS group. A previous study reported that increased activity in the right DLPFC is associated with state, but not with trait anxiety (Dresler et al., 2009). Therefore, this result may reflect participant temporal anxiety due to the unfamiliar cognitive task and experimental environment. However, in this study, the gross BDI score and trait anxiety score were significantly higher in the BIS than in the BAS group, and state anxiety had a non-significant correlation with right DLPFC activity in both the stimuli with negative valence and stimuli with positive valence conditions (negative: *r* = −0.21, *p* = 0.27; positive *r* = 0.06, *p* = 0.74, see Supplementary Figure 3). Thus, the increased activity in the right DLPFC in the stimuli with positive valence condition did not depend on participant anxiety due to the unfamiliar cognitive task and experimental environment but may have depended on the personality traits. Moreover, the right lateral prefrontal cortex is an important area related to the removal of distractors (Sawamura et al., 2014). The increased Oxy-Hb concentration in the right DLPFC in the BIS group in the positive and negative valence conditions may have reflected the intensity of distraction caused by both emotional valence and emotional arousal. Additionally, a meta-analysis revealed that higher right DLPFC activity was found in male than in female participants in all emotional valence conditions (Stevens and Hamann, 2012). Thus, sex differences could have also contributed to the differences between the present results and those of previous studies.

Conversely, the right DLPFC Oxy-Hb level was significantly lower in the BAS than in the BIS group in the stimuli with positive valence condition, and lower bilateral DLPFC activity was observed in the BAS group in all emotional valence conditions, which differed from the results of previous studies. Previous studies used verbal *n*-back tasks, involving left-specific lateralization of the DLPFC (Smith and Jonides, 1999; Baddeley, 2003). Therefore, the combination of the verbal *n-back* task with positive stimuli in previous studies could have induced significant left DLPFC activity increases.

Finally, this study used a cognitive task designed to examine hemisphere-specific DLPFC activity. Regarding the results, no increase in Oxy-Hb concentration in the left DLPFC could be found. A previous study investigating the relationship between resting-state brain activity and personality traits using the BIS/BAS scale reported that there was a significant relationship between the BIS score and right frontal brain activity, but not between the BAS score and the left frontal brain activity (Neal and Gable, 2017). Some studies have classified the BAS into more detailed and precise traits and have revealed that a part of those traits is related to resting-state left frontal brain activity (Coan et al., 2006; Gable and Poole, 2014). Accordingly, the gross BAS score was used, which would have included numerous personality trait aspects. Moreover, the relationship between the BAS and left frontal brain activity would be considered to depend on participant sensitivity to the stimuli (Neal and Gable, 2017). Therefore, the lower activity in the bilateral DLPFC in the BAS group may have been caused by low sensitivity to emotional stimuli, depending on participant motivation to the stimuli.

The working memory network in the prefrontal cortex is related to auditory-verbal and visuospatial working memory function (Klingberg, 2006; Takeuchi et al., 2011). However, a relationship between task performance and DLPFC activity could not be found. These results were consistent with those of previous studies (Kopf et al., 2013; Ozawa et al., 2014; Ozawa and Hiraki, 2017; Ozawa et al., 2019). The individual differences in task performance could have affected inconsistent task performance and DLPFC activity. From a different perspective, task difficulty could have also affected these results. In this study, the dual *n*-back tasks had high difficulty, and this may have partly caused decreased activity in the PFC (Klingberg, 2009; Gilbert et al., 2012).

### Limitations

There are several limitations to this study. The first pertains to differences in individual ability to solve the task. A previous study reported that the effect of emotional stimuli was more evident when task performance was analyzed by separating high and low-performance groups (Gray, 2001). However, the present sample size was insufficient to analyze this possibility. Also, the sample size was estimated based on the result of the interaction of working memory task performance between group and emotional valence as the primary endpoint; therefore, the sample size for the correlation analysis in each group could not have sufficed. The second limitation pertains to the spatial resolution of NIRS. Precise brain areas could not be defined because of NIRS’ low spatial resolution, and the subcortical areas could not be examined. However, attempts were made to improve the spatial accuracy of the DLPFC, which is engaged in working memory performance. Moreover, differences in local activity within the DLPFC area could not be determined. The third limitation pertains to the lack of deoxygenated hemoglobin (Deoxy-Hb) data. Whilst it has been argued that Oxy-Hb is the most reliable measure of cerebral blood flow (Hoshi et al., 2001; Strangman et al., 2002), the lack of Deoxy-Hb data comprises a limitation of this study and should be noted. For instance, it has been argued that Deoxy-Hb is most similar to the blood oxygen-dependent response in functional magnetic resonance imaging, and the inclusion of both Oxy-Hb and Deoxy-Hb data would have allowed better physiological interpretations (Tachtsidis and Scholkmann, 2016). Finally, the classification method of personality traits was also a limitation. In this study, personality traits were simply classified based on the BIS/BAS scale. Trait dimensions vary according to the personality trait scales used. Only two general motivational systems that underlie behavior based on the reinforcement sensitivity theory of personality were targeted in this study. Therefore, evaluating different dimensions of personality traits would be required.

### Conclusion

In this study, the influence of personality traits on the impact of emotional stimuli was examined focusing on *n*-back task performance and brain activity changes. Task performance showed a significantly increased RT in the negative valence condition independent of personality traits. The results of Oxy-Hb changes showed a significant interaction between personality traits and emotional valence. Further subgroup analysis of each hemisphere revealed that the right DLPFC activity was significantly higher in the negative valence than in the neutral valence condition in the BIS group; the right DLPFC activity was also significantly higher in the BIS group than in the BAS group in the positive valence condition. This result is considered to support the hypothesis that the impact of emotional stimuli on activity in the DLPFC is affected by personality traits. The results also suggested that it would be important to consider personality traits when examining the impact of emotional stimuli. Further studies with large sample sizes will be needed to examine differences in the influence emotional stimuli exert on working memory performance, considering the effect of personality traits to better understand individual differences pertaining to the impact of emotional stimuli.

## Data Availability Statement

The datasets generated during and/or analyzed during the current study are available from the corresponding author on reasonable request.

## Ethics Statement

The studies involving human participants were reviewed and approved by the Ethics Committee of the Health Sciences University of Hokkaido. The patients/participants provided their written informed consent to participate in this study.

## Author Contributions

MS and DS: study conception and design. MS, SSaku, HS, MM, SY, and DS: acquisition of data. MS, SSaku, and DS: analysis and interpretation of data and critical revision. MS, SSaku, MM, SY, TK, SSaka, and DS: drafting of the manuscript. All authors (MS, SSaku, HS, MM, SY, TK, SSaka, and DS): final approval of the article.

## Conflict of Interest

The authors declare that the research was conducted in the absence of any commercial or financial relationships that could be construed as a potential conflict of interest.

## Abbreviations

BDI: Beck Depression Inventory
DLPFC: dorsolateral prefrontal cortex
ER: error rate
IAPS: International Affective Picture System
NIRS: near-infrared spectroscopy
ROI: region of interest
RT: reaction time
STAI: State-Trait Anxiety Inventory.
