# Supplementary material for: Personality Traits Modulate the Impact of Emotional Stimuli During a Working Memory Task: A Near-Infrared Spectroscopy Study
Source: Front Behav Neurosci. 2020 Sep 17;14:514414. doi: 10.3389/fnbeh.2020.514414 (PMC7528631; doi:10.3389/fnbeh.2020.514414)
Supplement: Supplementary file 1 [file Data_Sheet_1.docx]

Personality Traits Modulate the Impact of Emotional Stimuli During a Working Memory Task: A Near-infrared Spectroscopy Study

# Supplementary Figures and Tables

## Supplementary Figures

## Supplementary Tables


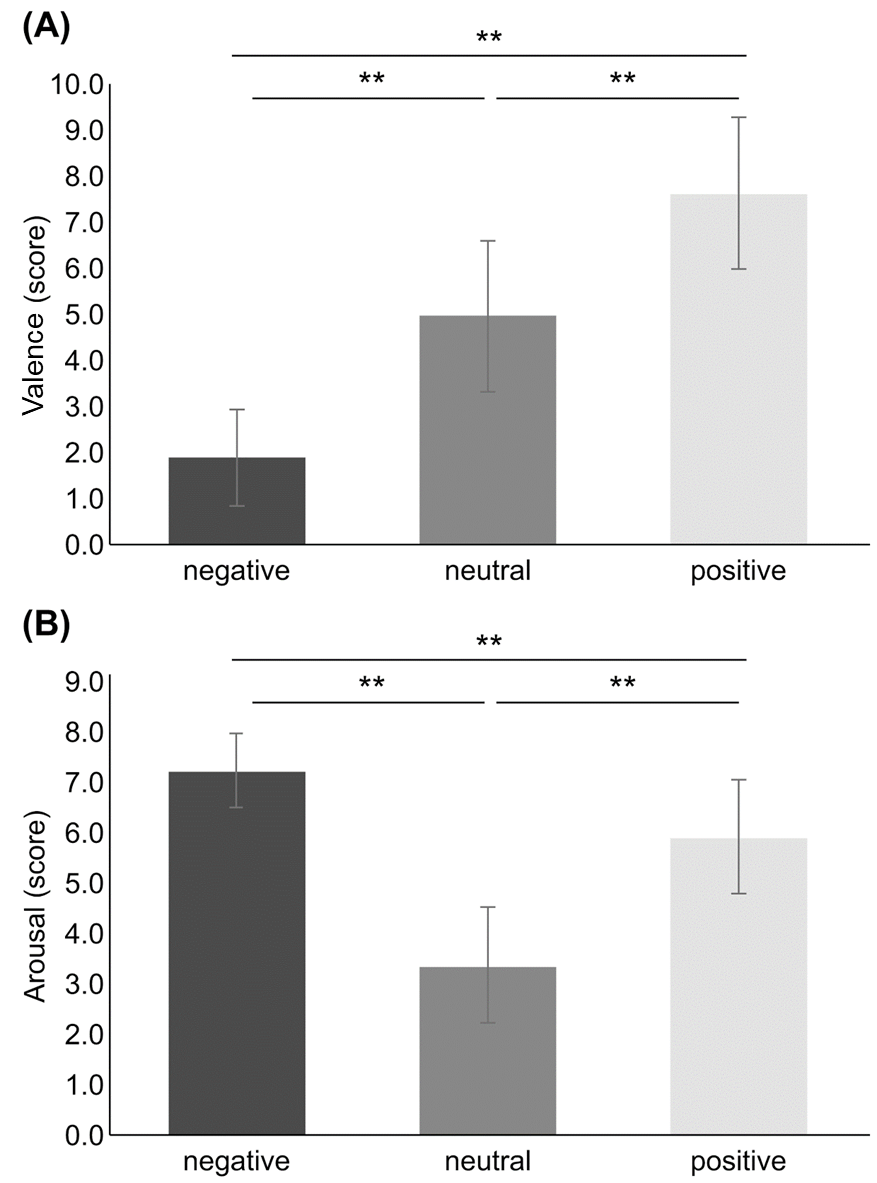


**Supplementary Figure 1. Comparison of emotional valence rating and arousal rating among the three emotional valences.** (A) Comparison of emotional valence rating among the three emotional valences. (B) Comparison of arousal rating among the three emotional valences. **p* < 0.01; ***p* < 0.001. Error bars indicate standard error.





**Supplementary Figure 2.** 25 participants were included in SCR analysis because 5 participants were excluded by missing data due to device malfunction. The averaged SCR during picture presentation periods was significantly higher than those during rest periods in every emotional valence (*p*＜0.01). **p* < 0.01. Error bars indicate standard error.


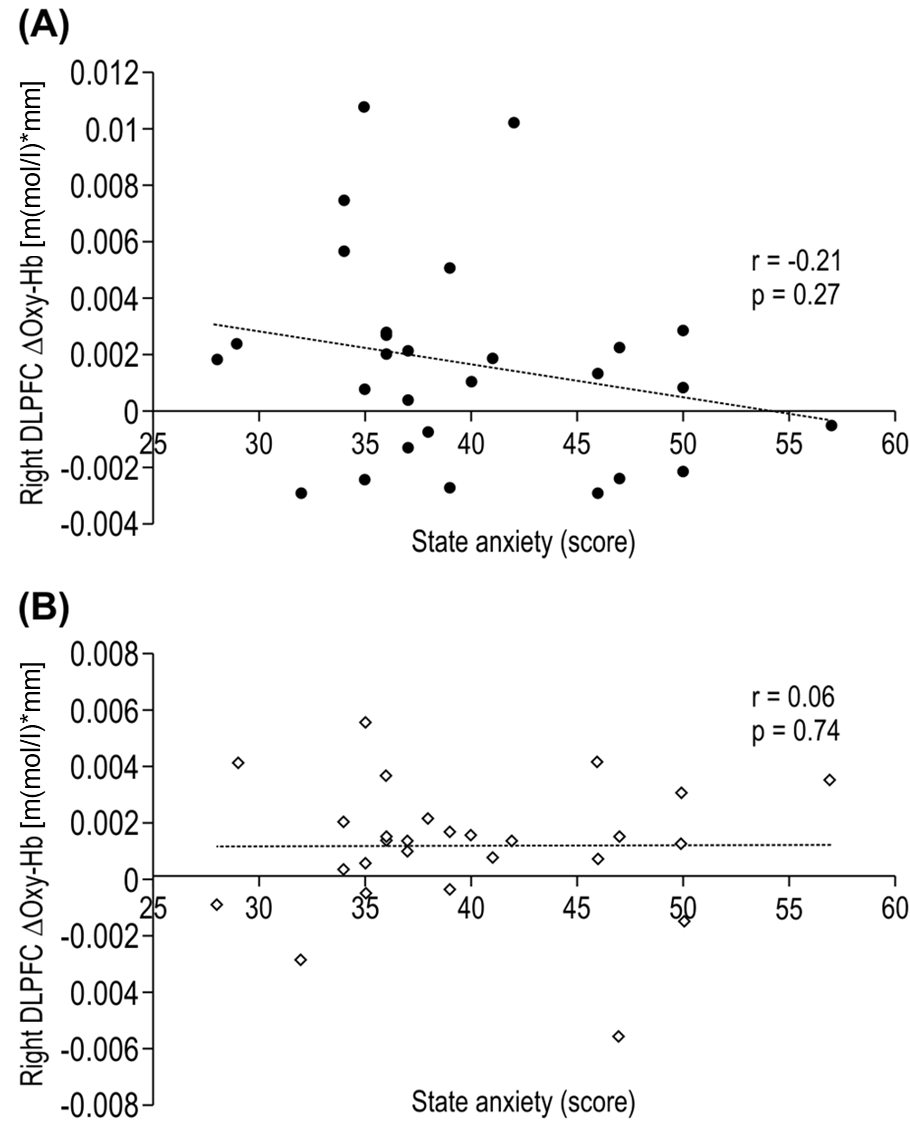


**Supplementary Figure 3. Correlation between state anxiety score and Right DLPFC ΔOxy-Hb.** No significant correlations were shown in negative valence (A) and positive valence (B).


Supplementary Table

**Supplementary Table 1. Emotional images of IAPS selected in the preliminary experiment**

| Image | Number | Valence | Arousal |
| --- | --- | --- | --- |
| Controls for the scaling criteria |  |  |  |
| Burn Victim (as negative) | 3100 | 1.60 | 6.49 |
| Basket (as neutral) | 7010 | 4.94 | 1.76 |
| Family (as positive) | 2154 | 8.03 | 4.48 |
| Negative images |  |  |  |
| Burn Victim* | 3053 | 1.31 | 6.91 |
| Headless Body | 3001 | 1.62 | 6.64 |
| Mutilation* | 3069 | 1.70 | 7.03 |
| Vomit | 9325 | 1.89 | 6.01 |
| Cat | 9571 | 1.96 | 5.64 |
| Neutral images |  |  |  |
| Mug | 7009 | 4.93 | 3.01 |
| Girl* | 2411 | 5.07 | 2.86 |
| Outlet | 6150 | 5.08 | 3.22 |
| Shadow | 2880 | 5.18 | 2.96 |
| Bridge* | 7547 | 5.21 | 3.18 |
| Positive images |  |  |  |
| Couple | 2530 | 7.80 | 3.99 |
| Children* | 2347 | 7.83 | 5.56 |
| Family* | 2340 | 8.03 | 4.90 |
| Sea | 5825 | 8.03 | 5.46 |
| Puppies | 1710 | 8.34 | 5.41 |
|  | |  |  |

*Images selected in the preliminary experiment and used in the main experiment.

IAPS: International Affective Picture System

**Supplementary Table 2. Montreal Neurological Institute coordinate data in all channels**

| Channel number | x | y | z |
| --- | --- | --- | --- |
| 1 | -44.96 | 47.54 | 22.18 |
| 2 | -27.54 | 49.79 | 39.18 |
| 3 | -51.75 | 36.89 | 20.46 |
| 4 | -38.71 | 38.71 | 40.86 |
| 5 | -17.21 | 41.00 | 53.43 |
| 6 | -46.50 | 28.86 | 40.79 |
| 7 | -28.75 | 29.68 | 55.57 |
| 8 | -54.54 | 16.18 | 36.11 |
| 9 | -41.21 | 18.29 | 56.93 |
| 10 | -19.07 | 20.29 | 66.79 |
| 11 | -49.29 | 6.36 | 52.50 |
| 12 | -31.39 | 8.79 | 66.14 |
| 13 | -60.07 | -8.71 | 44.68 |
| 14 | -42.36 | -5.57 | 62.96 |
| 15 | -19.89 | -3.61 | 74.39 |
| 16 | -54.25 | -19.36 | 57.64 |
| 17 | -33.50 | -16.71 | 73.00 |
| 18 | -63.61 | -33.86 | 46.11 |
| 19 | -45.93 | -29.29 | 66.54 |
| 20 | -22.11 | -26.68 | 76.04 |
| 21 | -55.00 | -44.71 | 54.75 |
| 22 | -34.89 | -41.04 | 71.18 |
| 23 | 32.71 | 48.75 | 38.86 |
| 24 | 47.86 | 46.64 | 24.29 |
| 25 | 23.25 | 40.54 | 52.89 |
| 26 | 42.25 | 37.96 | 41.29 |
| 27 | 54.43 | 35.96 | 23.89 |
| 28 | 33.29 | 29.25 | 54.61 |
| 29 | 49.11 | 28.82 | 41.75 |
| 30 | 23.86 | 20.71 | 65.29 |
| 31 | 43.29 | 19.46 | 55.71 |
| 32 | 56.32 | 14.86 | 38.64 |
| 33 | 33.64 | 10.11 | 64.82 |
| 34 | 50.68 | 7.11 | 53.25 |
| 35 | 22.21 | -1.89 | 73.57 |
| 36 | 43.86 | -4.04 | 63.32 |
| 37 | 60.43 | -7.68 | 47.79 |
| 38 | 34.61 | -15.36 | 72.68 |
| 39 | 53.68 | -17.61 | 59.79 |
| 40 | 23.46 | -26.07 | 76.43 |
| 41 | 45.50 | -26.54 | 67.43 |
| 42 | 63.54 | -31.54 | 50.32 |
| 43 | 34.04 | -38.43 | 72.39 |
| 44 | 54.46 | -41.14 | 57.25 |

**Supplementary Table 3. Correlation analysis between the BIS/BAS scores and behavioral data and DLPFC activity**

|  |  | BIS score | |  | BAS score | |  | ER | |  | RT | |
| --- | --- | --- | --- | --- | --- | --- | --- | --- | --- | --- | --- | --- |
|  |  | *r* | *p* |  | *r* | *p* |  | *r* | *p* |  | *r* | *p* |
| Dual 2-back task | (*n* = 30) |  |  |  |  |  |  |  |  |  |  |  |
| ER | Negative | -0.042 | 0.828 |  | -0.001 | 0.995 |  |  |  |  |  |  |
|  | Neutral | -0.002 | 0.994 |  | 0.199 | 0.292 |  |  |  |  |  |  |
|  | Positive | 0.026 | 0.891 |  | -0.059 | 0.758 |  |  |  |  |  |  |
| RT | Negative | 0.053 | 0.783 |  | 0.066 | 0.729 |  |  |  |  |  |  |
|  | Neutral | -0.027 | 0.887 |  | 0.111 | 0.560 |  |  |  |  |  |  |
|  | Positive | -0.104 | 0.585 |  | -0.027 | 0.885 |  |  |  |  |  |  |
|  |  |  |  |  |  |  |  |  |  |  |  |  |
| DLPFC activity | (*n* = 28) |  |  |  |  |  |  |  |  |  |  |  |
| Left | negative | -0.045 | 0.820 |  | -0.180 | 0.359 |  | 0.161 | 0.414 |  | 0.107 | 0.588 |
|  | neutral | -0.101 | 0.610 |  | 0.167 | 0.395 |  | 0.301 | 0.119 |  | 0.232 | 0.235 |
|  | positive | 0.031 | 0.876 |  | -0.019 | 0.925 |  | 0.048 | 0.810 |  | 0.105 | 0.593 |
| Right | negative | 0.173 | 0.379 |  | -0.068 | 0.733 |  | 0.095 | 0.631 |  | 0.043 | 0.828 |
|  | neutral | -0.004 | 0.983 |  | 0.131 | 0.506 |  | -0.273 | 0.160 |  | -0.245 | 0.209 |
|  | positive | 0.192 | 0.328 |  | -0.192 | 0.328 |  | -0.052 | 0.794 |  | 0.037 | 0.851 |

BIS: behavioral inhibition system, BAS: behavioral activation system, DLPFC: dorsolateral prefrontal cortex, ER: error rate, RT: reaction time
